# Correspondence: On the enzymology and significance of HSPA1 lysine methylation

**DOI:** 10.1038/ncomms11464

**Published:** 2016-06-20

**Authors:** Magnus E. Jakobsson, Anders Moen, Pål Ø Falnes

**Affiliations:** 1 Department of Biosciences, University of Oslo, P.O. BOX 1066, Blindern, Oslo 0316, Norway

Cho *et al.*^1^ recently reported that the methyltransferase SETD1A catalyses dimethylation of the stress-inducible Hsp70 protein HSPA1 at lysine 561 (HSPA1-K561me2), while we^2^ and others^3^ have later reported that a different enzyme, METTL21A, methylates HSPA1-K561 in vitro and in vivo. Moreover, Cho *et al.*^1^ reported that HSPA1-K561me2 specifically localizes to the cell nucleus, where it activates Aurora kinase B, and that this modification is increased in various cancers. Here, we present data that conflict with the conclusions drawn by Cho *et al*.^1^, indicating that SETD1A does not play a direct role in HSPA1-K561 methylation and that HSPA1-K561me2 is not specifically localized to the nucleus.

To further investigate the roles of human METTL21A and SETD1A in HSPA1-K561 methylation *in vivo*, the HSPA1-K561 methylation status in a METTL21A knockout cell line was assessed by mass spectrometry. HSPA1-K561 was found to be mostly trimethylated (HSPA1-K561me3) in wild-type KBM-7 cells (Fig. 1a), a methylation pattern similar to what we previously observed in human cell lines and cancer samples^2^,^4^. In contrast, HSPA-K561 was exclusively unmethylated in the corresponding METT21A knockout cells, which expressed SETD1A (Fig. 1a). This shows that METTL21A is required for HSPA1-K561 methylation, and suggests no involvement of SETD1A. However, Cho *et al.*^1^ suggested that SETD1A specifically mediates the formation of the dimethylated species, HSPA1-K561me2, and previous *in vitro* experiments, using high enzyme concentrations, have only demonstrated METTL21A-mediated formation of HSPA1-K561me3. Thus, the possibility existed that METTL21A is a processive enzyme responsible only for the formation of HSPA1-K561me3, whereas SETD1A is involved in introducing lower methylation states, such as HSPA1-K561me2. To further explore this, we incubated recombinant HSPA1 with varying amounts of METTL21A *in vitro*, and, subsequently determined the HSPA1-K561 methylation status by protein mass spectrometry. Clearly, HSPA1 incubated with intermediate amounts of METTL21A displayed a mixture of the three methylated forms (me1, me2 and me3), demonstrating that METTL21A is a non-processive enzyme capable of generating all methylation states on HSPA1-K561 (Fig. 1b). Cho *et al.*^1^ observed alterations in HSPA1 methylation upon modulating SETD1A levels by knockdown and overexpression. However, as SETD1A is already established as a histone methyltransferase that mono-, di- and trimethylates lysine 4 in histone H3 (ref. 5), and thereby regulates gene expression^6^, the likely possibility exists that the observed effects were indirect and not reflecting SETD1A-mediated HSPA1 methylation. Moreover, Cho *et al.*^1^ presented no biochemical evidence that SETD1A can catalyse HSPA1-K561 methylation *in vitro*. Therefore, the above data, taken together with the published literature^2^,^3^ on METTL21A-mediated HSPA1 methylation, strongly indicates that METTL21A is the sole enzyme responsible for methylation of HSPA1-K561.

Cho *et al.*^1^ reported that HSPA1-K561me2 localizes predominantly to the nucleus of cancer cells. Using subcellular fractionation in combination with protein mass spectrometry, we have here further investigated the reported methylation-dependent nuclear localization of HSPA1. We observed, in agreement with Cho *et al.*^1^ and the published literature^7^, HSPA1 in both the cytosolic and nuclear fractions (Fig. 2). Notably, we found, using two different cell lines, HeLa and HEK-293, the HSPA1-K561 methylation pattern to be indistinguishable between the nuclear and cytosolic fractions, with HSPA1-K561me3 as the predominant form (Fig. 2). The nuclear accumulation of HSPA1 reported by Cho *et al.*^1^ was entirely based on nuclear immunostaining observed with a HSPA1-K561me2-specific antibody, and the above results suggest to us that this antibody, despite apparently recognizing HSPA1-K561me2 in western blotting, recognizes a nuclear protein other than HSPA1 when used for immunofluorescence and imaging. Cho *et al.*^1^ further reported that HSPA1-K561me2, compared to unmethylated HSPA1, was a stronger activator of Aurora kinase B, thereby linking HSPA1-K561me2 to cell proliferation. However, we question these conclusions, since they largely rely upon the assumptions that HSPA1-K561 is dimethylated in the nucleus and unmethylated in the cytoplasm, and that differences between wild-type HSPA1 and a K561R mutant with respect to Aurora kinase B binding and activation reflect HSPA1-K561me2-dependent effects. In our opinion, these reported effects are likely caused by the mutation of a highly conserved residue (the residue corresponding to K561 is conserved in most eukaryotic and prokaryotic Hsp70 homologues), and not related to HSPA1 methylation.

In summary, we have here presented data and arguments that put into question the role of SETD1A in mediating the methylation of HSPA1 and question whether HSPA1-K561me2 is specifically localized to the nucleus.

## Methods

### Cell culture and preparation of cellular protein fractions

KBM-7 wild type (P00174R07) and METTL21A-gene-trapped cells (P00213G12) were purchased from Horizon Genomics (formerly Haplogen); HeLa cells were obtained from ATCC and HEK-293 cells (Flp-In T-REx 293) from Invitrogen. All cell lines were tested negative for *Mycoplasma* infection. KBM-7 cells were cultured in IMDM-Glutamax^TM^ (Thermo Scientific), while HeLa and HEK-293 cells were cultured in DMDM-Glutamax^TM^ (Thermo Scientific). All media was supplemented with 10% fetal bovine serum and 100 U ml^−1^ penicillin/streptomycin. Whole-cell extracts for western blot analysis of METTL21A and SETD1A protein levels were prepared by culturing cells to near confluency in a 10-cm-diameter dish, whereafter cells were harvested by scraping and collected by centrifugation at 300*g* for 5 min. Cells were thereafter washed three times by dilution in 1 ml PBS and collected by centrifugation as described above. The resulting pellet was dissolved in 1% SDS, 1 mM phenylmethylsulphonyl fluoride (PMSF) (Sigma-Aldrich) and 1x protease inhibitor cocktail (Sigma-Aldrich), whereafter the protein concentration was determined with the BCA method.

Subcellular fractions were prepared using a slightly modified version of the so-called rapid, efficient, and practical (REAP) method^8^. In brief, HeLa and HEK-293 cells were cultured to near confluency on 10-cm-diameter dishes. The cells were then washed with ice-cold PBS (pH 7.4), harvested by scraping, collected in 1 ml cold PBS and pelleted by centrifugation at 300*g* for 5 min. The pellet was resuspended in 900 μl lysis buffer (PBS supplemented with 0.1% NP40, PMSF (Sigma-Aldrich) and protease inhibitor cocktail (Sigma-Aldrich)), whereafter a 300-μl sample representing ‘whole-cell lysate' was removed for later analysis. The remaining sample was processed in a pop-spin centrifuge for 30 s, whereafter 300 μl of the supernatant, representing ‘cytosolic fraction', was removed. The remaining supernatant was then removed and the pellet, representing ‘nuclear fraction', was washed three times in lysis buffer and harvested by centrifugation as above. All fractions were then denatured in NuPAGE (Invitrogen) buffer and a normalized amount of sample was loaded on SDS-polyacrylamide gel electrophoresis (PAGE) for western blot and mass spectrometry analyses.

### Western blotting

Twenty micrograms of protein extracts from KBM-7 cells and 30 μl ‘whole-cell extract', and equivalent amount of cytosolic and nuclear fractions, from HeLa and HEK-293 cells were separated by SDS-PAGE and transferred to polyvinylidene difluoride membranes. Membranes were blocked overnight with 5% bovine serum albumin in Tris-buffered saline (TBS; pH 7.4), incubated with relevant primary antibodies (see below), washed three times with TBS for 10 min, incubated with appropriate horseradish peroxidase-coupled secondary antibody and finally washed six times for 10 min with TBS supplemented with 0.05% Tween. Membranes were then treated with SuperSignal™ enhanced chemiluminescent substrate (Thermo Fisher) and staining was visualized with a CCD-based imager. The following primary antibodies were used (working dilution is indicated): anti-HSPA1 (Abcam ab79852; 1:10,000), anti-METTL21A (Sigma-Aldrich HPA034712; 1:250), anti-SETD1A (Abcam ab70378; 1:1,000), anti-beta Actin (Abcam ab8227; 1:3,000), anti-alpha Tubulin (Abcam ab4074; 1:300) and anti-Histone H3 (Abcam ab1791; 1:3,000).

### Mass spectrometry analysis

Analysis of HSPA1-Lys561 methylation events was essentially performed as previously described^2^,^4^. In brief, protein samples were separated by SDS-PAGE, whereafter the gel region encompassing HSPA1 was excised and treated with the endoprotease AspN. The resulting peptides were analysed by reverse-phase liquid chromatography coupled to a LTQ Orbitrap XL mass spectrometer (Thermo Scientific) via nanoelectrospray, using collision-induced fragmentation. Ion chromatograms corresponding to the different methylated forms of HSPA1-Lys561 were generated by gating for relevant mass-to-charge ratios of the AspN-proteolytic peptide Asp555-Ala565, *z*=2, of HSPA1 using Xcalibur Qual Browser (v2.0.7). The selective ion settings used were *m*/*z*=573.8037 (me0), 580.8115 (me1), 587.8193 (me2) and 594.8272 (me3)+/−10 p.p.m. The relative abundance of the different methylated species of HSPA1-Lys561 was approximated as the ratio between the area under the relevant chromatographic peak (for example, MeO, Me1, Me2 or Me3) and the sum of the area under all peaks (MeO, Me1, Me2 and Me3). The area under the peaks was determined by integration using Xcalibur Qual Browser (v2.0.7).

### Expression and purification of recombinant proteins

The previously described^2^ plasmids pET28a-METTL21A and pGEX-6p-HSPA1 were transfected into the *Escherichia coli* BL21-CodonPlus(DE3)-RIPL expression strain (Agilent). 6xHis tagged METTL21A and GST-tagged HSPA1 were thereafter purified using Ni-NTA agarose (Qiagen) and Glutathione Sepharose 4B (GE Healthcare), respectively, according to the manufacturer's instructions. After affinity purification the buffer was changed to 20 mM Tris (pH 6.8), 100 mM Nacl and 1 mM DTT by sequential dilution and concentration using Vivaspin 20 ultracentrifugation columns with a molecular weight cutoff of 10 kDa (for METTL21A) or 50 kDa (for HSPA1) (Sartorius AG). Proteins were then aliquoted and stored at −80 °C and the concentration was determined using the BCA method.

## Additional information

**How to cite this article:** Jakobsson, M. E. *et al.* Correspondence: On the enzymology and significance of HSPA1 lysine methylation. *Nat. Commun.* 7:11464 doi: 10.1038/ncomms11464 (2016).

## Figures and Tables

**Figure 1:**
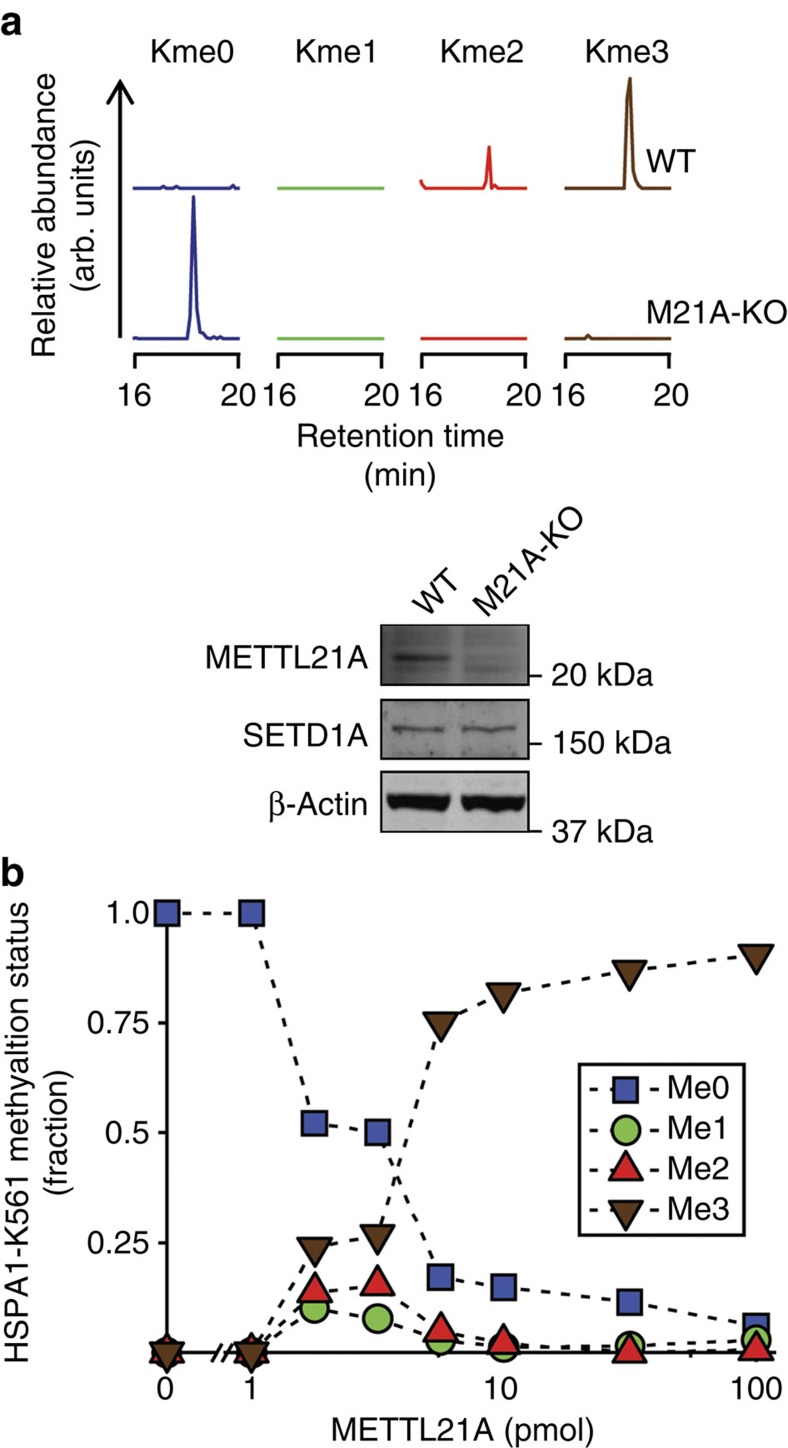
METTL21A-mediated methylation of HSPA1-K561. (**a**) METTL21A knockout abrogates methylation of HSPA1-K561 *in vivo*. Top panel, extracted ion chromatograms corresponding to mass-to-charge ratios of the various methylated forms (Kme0, Kme1, Kme2 and Kme3) of a previously studied^2^,^4^ AspN-generated proteolytic peptide covering Asp555–Ala565 of HSPA1 from KBM-7 wild-type (WT) and corresponding METTL21A knock-out (M21A-KO) cells. Bottom panel, western blot analysis of METTL21A and SETD1A in cell lines analysed in the upper panel. β-Actin was used as a loading control. (**b**) METTL21A-mediated methylation of HSPA1-K561 is non-processive. Recombinant human HSPA1 was treated with varying amounts of METTL21A in the presence of the methyl donor *S*-adenosyl methionine and the relative abundance of the various methylated forms of K561 was determined by mass spectrometry.

**Figure 2:**
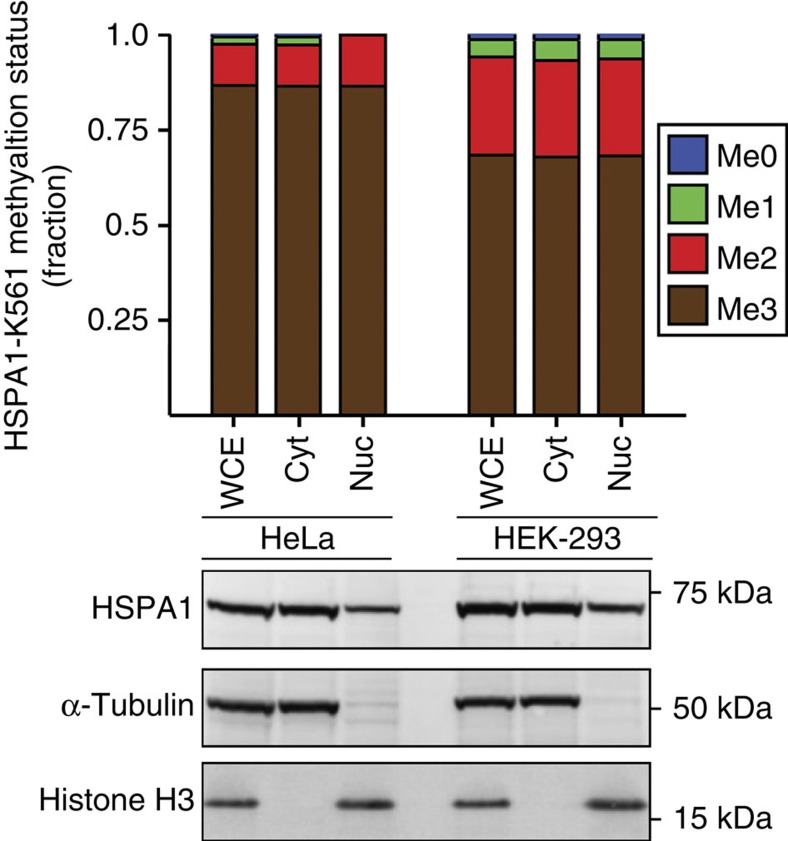
Methylation status of HSPA1-K561 in subcellular fractions. Top panel, methylation status in whole-cell extracts (WCE) as well as in cytosolic (Cyt) and nuclear (Nuc) fractions from HeLa and HEK-293 cells was determined by mass spectrometry as in Fig. 1a. Bottom panel, western blot analysis of total HSPA1, alpha-tubulin (cytosolic marker) and histone H3 (nuclear marker) in protein samples analysed in the upper panel.

## References

[b1] Cho H. S. *et al.* Enhanced HSP70 lysine methylation promotes proliferation of cancer cells through activation of Aurora kinase B. Nat. Commun. 3, 1072 (2012). 22990868 10.1038/ncomms2074 PMC3658001

[b2] Jakobsson M. E. *et al.* Identification and characterization of a novel human methyltransferase modulating Hsp70 function through lysine methylation. J. Biol. Chem. 288, 27752–27763 (2013). 23921388 10.1074/jbc.M113.483248 PMC3784692

[b3] Cloutier P., Lavallee-Adam M., Faubert D., Blanchette M. & Coulombe B. A newly uncovered group of distantly related lysine methyltransferases preferentially interact with molecular chaperones to regulate their activity. PLoS Genet. 9, e1003210 (2013). 23349634 10.1371/journal.pgen.1003210 PMC3547847

[b4] Jakobsson M. E., Moen A., Davidson B. & Falnes P. O. Hsp70 (HSPA1) lysine methylation status as a potential prognostic factor in metastatic high-grade serous carcinoma. PLoS ONE 10, e0140168 (2015). 26448330 10.1371/journal.pone.0140168 PMC4598032

[b5] Shinsky S. A., Monteith K. E., Viggiano S. & Cosgrove M. S. Biochemical reconstitution and phylogenetic comparison of human SET1 family core complexes involved in histone methylation. J. Biol. Chem. 290, 6361–6375 (2015). 25561738 10.1074/jbc.M114.627646 PMC4358272

[b6] Deng C. *et al.* USF1 and hSET1A mediated epigenetic modifications regulate lineage differentiation and HoxB4 transcription. PLoS Genet. 9, e1003524 (2013). 23754954 10.1371/journal.pgen.1003524 PMC3675019

[b7] Daugaard M., Rohde M. & Jaattela M. The heat shock protein 70 family: highly homologous proteins with overlapping and distinct functions. FEBS Lett. 581, 3702–3710 (2007). 17544402 10.1016/j.febslet.2007.05.039

[b8] Suzuki K., Bose P., Leong-Quong R. Y., Fujita D. J. & Riabowol K. REAP: a two minute cell fractionation method. BMC Res. Notes 3, 294 (2010). 21067583 10.1186/1756-0500-3-294 PMC2993727

